# Clinical review and analysis of artery of Percheron infarction

**DOI:** 10.1016/j.ibneur.2023.04.006

**Published:** 2023-05-27

**Authors:** Jing Li, Junling Ge, Shuhui Yang, Guoen Yao

**Affiliations:** a Departments of Neurology, the Frist center of Chinese PLA General Hospital, China; b Departments of Radiology, Chinese PLA General Hospital, 51 Fucheng road, Haidian District, 100048 Beijing, China

**Keywords:** Artery of Percheron, Stroke, Bilateral medial thalamic stroke, Ischemic cerebrovascular disease

## Abstract

Artery of Percheron infarction is a rare one of the neurovascular structure variants of acute ischemic stroke characterized by bilateral paramedian thalamic infarcts (BTPI), with or without mesencephalic infarction. Due to the low occurrence rate and various clinical manifestations, the early diagnosis of this disease is often missed. In addition, it is also difficult to diagnose this disease in an early implementation phase because cranial imaging and intracranial vascular imaging may show negative results. So far, its clinical cases have been rarely reported. We systematically reviewed the clinical manifestations, imaging characteristics, anatomical basis, and differentiation diagnosis of the artery of Percheron infarction and reported on three patients and their clinical and radiological medical imaging characteristic findings. We found that most of the infarct lesions in patients with an AOP infraction could not be displayed within a few hours or could not be fully displayed, even the embolism events, most of which showed typical imaging lesions at late review. The decrease of transient consciousness was obvious over the course of the three patients, and the decrease of active communication was also a major feature. Among the three cases, one patient had unilateral upper eyelid ptosis and miosis; the initial symptom of another patient was dizziness; and the other person had decreased computing power after infarction. These clinical symptoms are easily ignored in the diagnosis and treatment of patients with AOP infarction. Therefore, reporting the three clinical cases mentioned above will provide assistance for subsequent research by increasing clinical data.

## Introduction

The Artery of Percheron (AOP) is a rare form of the thalamoperforating artery that comes from the P1 segment of the posterior cerebral artery. Its blockage results in bilateral paramedian thalamic infarction (BTPI), also known as AOP infarction, which may or may not involve middle cerebral infarction. The clinical presentation of AOP infarction typically includes altered consciousness, supranuclear vertical gaze palsy, and memory impairment (Aaron et al., 2015). However, diagnosing the disease early and accurately is challenging due to the varying infarction ranges and clinical symptoms. Furthermore, AOP infarction clinical research is scarce. Early diagnosis of acute cerebral infarction is critical for selecting appropriate treatment options and determining the prognosis. The purpose of this retrospective case study is to investigate the clinical manifestations and imaging features of three patients who experienced AOP infarction and were hospitalized at the Fourth Medical Centre of the Chinese PLA General Hospital during the period spanning from June 2018 to April 2019.

### Case Presentation 1

**Fig. 1:**
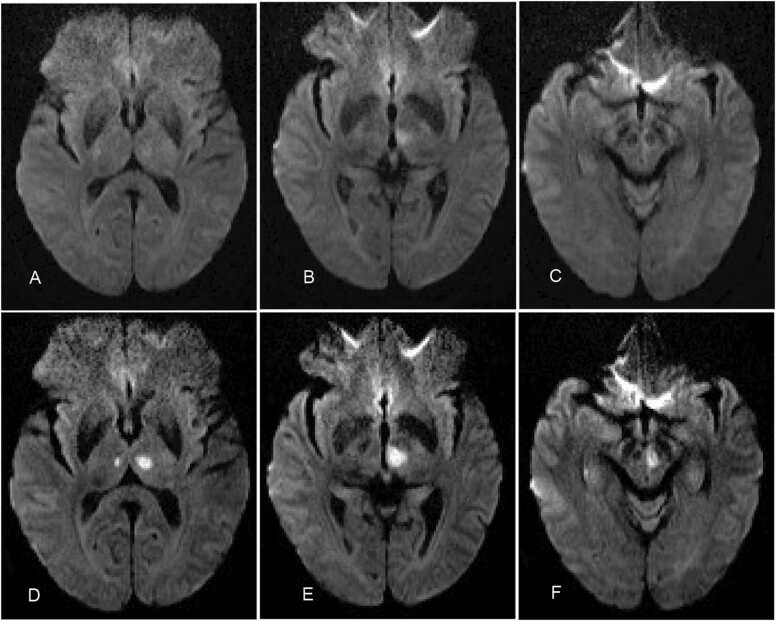
Diffusion-weighted MRI scans of Case 1 patient with AOP infraction. Axial diffusion-weighted images A–C showed high-intensity lesions in the paramedian region of the left thalamus seemed to involve the midbrain along the middle line; D–F reviewed clear diffusion-limited signals on the images in the left side of the midbrain and the paramedian thalamus after the third day after the onset of the disease.

A 72-year-old female patient was admitted to our emergency department with the sudden onset of unclear speech, impaired consciousness, and right-sided weakness. According to her family, she initially responded slowly and felt drowsy. She had trouble speaking after waking up. During her hospital stay, the patient gradually regained consciousness but remained lethargic. She had no trouble with basic skills testing or following directions, but she struggled with more complex tests of cortex function. Neuro-ophthalmological examination showed left upper eyelid ptosis, miosis, delayed response, right-sided weakness, and dysphonia. The patient's medical history included hypertension, diabetes mellitus, coronary heart disease, unstable angina pectoris, and atrial fibrillation (AFib) without anticoagulation after percutaneous coronary intervention (PCI). Brain magnetic resonance imaging (MRI) indicated an acute ischemia signal in the paramedian area of the left thalamus, which seemed to implicate the midbrain along the middle line with a reduced apparent diffusion coefficient (ADC) value and a strong signal on diffusion-weighted images (DWI) (Fig. 1A–C). The patient was determined to be ineligible for thrombolytic therapy due to exceeding the thrombolytic treatment window. The patient's modified Rankin score (mRS) was 2, and he was given anticoagulants and statins. After two days, the patient's level of awareness had significantly improved; nevertheless, a physical examination indicated that the patient coughed when drinking water and had superficial sensory disturbances on the left side. Up to that point, re-examined MRI images revealed fresh infarcts in the midbrain and bilateral parathalamic central areas (Fig. 1D–F). Magnetic resonance angiography (MRA) did not reveal any significant stenosis in the basilar artery or intracranial segment of the internal carotid artery, and the artery of Percheron was not visible. Due to a positive iodine allergy test, head and neck digital subtraction angiography (DSA) and computed tomographic arteriography (CTA) scans were not possible. Other cerebrovascular diseases, including tumors, rheumatic diseases, and hemological diseases, were excluded as the cause of the patient's sudden neurological deficits. Despite negative vascular imaging results, it was determined that the patient's lesions were caused by a disturbance in the blood supply region of the artery of Percheron.

### Case Presentation 2

**Fig. 2:**
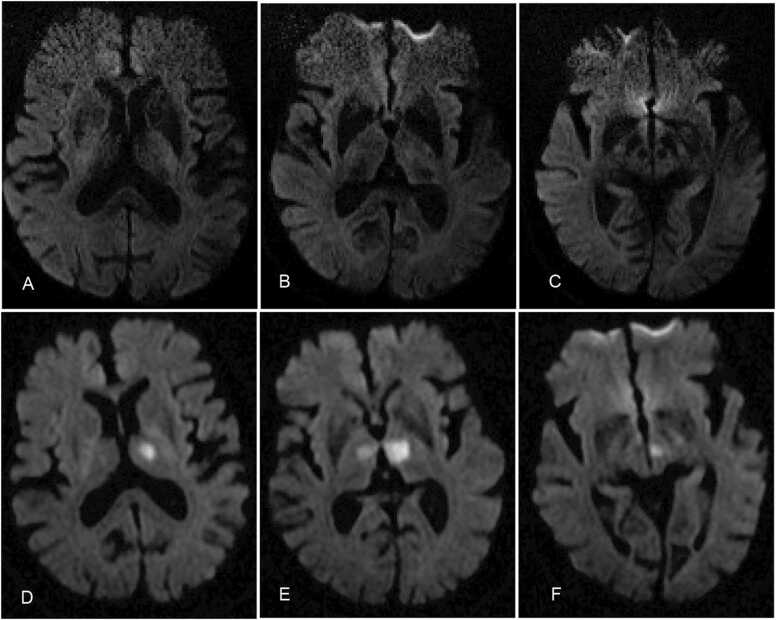
Diffusion-weighted MRI scans of Case 2 patient with AOP infraction. Diffusion weighted images A–C does not display abnormal diffusion-limited signals on initial presentation; D–F, images after review of increased disturbance of consciousness, showed hyperintense signals in the bilateral paramedian thalami and paramedian.

An 83-year-old male patient presented with paroxysmal vertigo, nausea, and tinnitus affecting both ears without hearing impairment. These symptoms occurred multiple times over several hours and lasted for tens of minutes each time, without any relation to changes in body position. The patient denied experiencing any symptoms of nervous system damage, such as weakness, numbness, abnormal sensations, acute visual changes, or walking deviation. A neurological examination revealed slow reaction times and recent memory impairment, but no other positive signs were found. Three days later, similar clinical symptoms reappeared, accompanied by increased sleep and reduced active communication, and the patient was re-admitted to our emergency department. The patient had a history of Kimura's disease (KD) for ten years, which he reported was under control. His past medical history also included hyperlipidemia, hypertension, and diabetes mellitus. A neurological examination on admission showed blurred speech, decreased computational power, poor understanding, a slow response, and bilateral pathological signs. At that time, the modified Rankin score (mRS) was 2. A brain computed tomography (CT) scan revealed no abnormal high-density shadows, and brain magnetic resonance imaging (MRI) taken on the same day showed no new abnormal signals (Fig. 2A–C). The carers reported that the patient had increased sleep and slower response times the next morning. The neurological examination revealed drowsiness, slower reaction times, decreased calculation and comprehension, and the rest of the examination was the same as before. The mRS score increased from 2 points to 3 points. Subsequent MRI confirmed bilateral paramedian thalamic infarction with midbrain involvement (Fig. 2D–F). The patient's family refused further examination, so head and neck vascular imaging could not be completed.

### Case Presentation 3

**Fig. 3:**
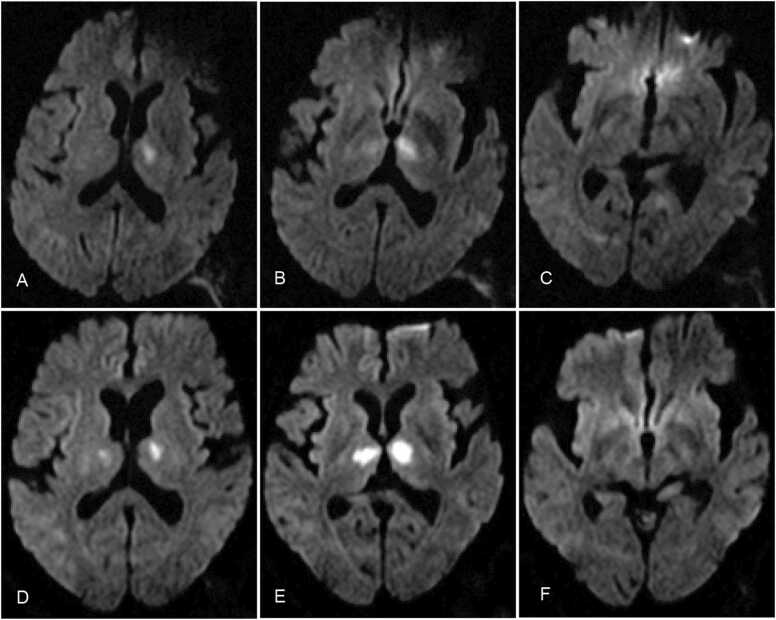
Diffusion-weighted MRI scans of Case 3 patient with AOP infraction. Diffusion weighted imaging A–C has shown asymmetric restricted diffusion in bilateral paramedian thalamus without midbrain 2 h after onset; D–F showed high signal intensity in the bilateral paramedian thalamus on DWI on the second day after onset.

**Fig. 4:**
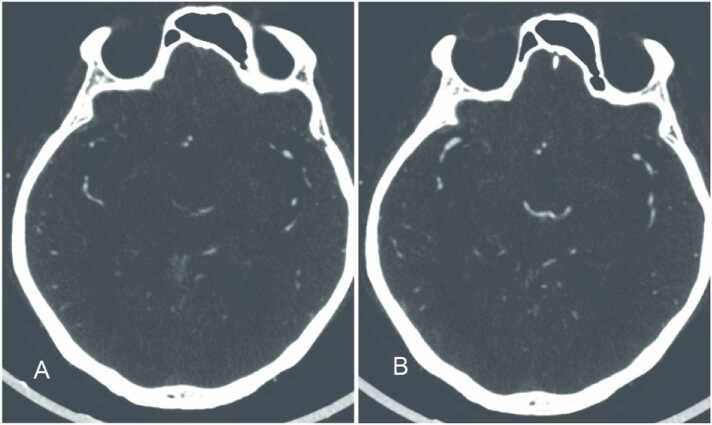
computed tomographic arteriography of Case 3 patient. CTA of Case 3 patient with AOP infraction show artery of Percheron.

A 59-year-old male patient presented suddenly with slurred speech and somnolence while watching TV. The patient's speech vagueness was mainly characterized by a slow speech rate, with no apparent abnormalities in speech content or grammar. The patient was able to answer questions correctly but responded slowly without active communication. Three weeks prior to the onset, the patient experienced transient dizziness symptoms but did not seek medical attention. The patient denied any history of hypertension, smoking, or coronary artery disease. A neurological examination revealed no obvious positive signs, except for somnolence and a slow speech rate. The patient had a modified Rankin Scale (mRS) score of 2 points at that time. Two hours after onset, brain MRI showed bilateral paramedian thalamic diffusion restriction without midbrain on DWI (Fig. 3A–C). There were slight asymmetry signals of the bilateral diffusion restriction, with the left area being relatively more affected than the right. Due to a history of black stool in the recent stage of medical consultation, intravenous thrombolytic therapy was contraindicated. On the second day of admission, the patient developed new signs of cognitive decline, which were observed during the neurological examination. The mRS score remained at 2 points. A repeat MRI showed symmetrically located lacunar infarcts within the paramedian vascular territories of both thalami on the following day (Fig. 3D–F). Head and neck CTA with contrast revealed mild degrees of atherosclerosis in the patient's intracranial arteries and vertebrobasilar artery, but no significant stenosis was observed. Additionally, the artery of Percheron was visualized (Fig. 4). The patient had impaired glucose tolerance, which was considered a risk factor for infarction. Other potential causes, such as heart disease, hypertension, cancer, rheumatism, and infection, were excluded. Based on the patient's clinical symptoms and imaging features, the diagnosis of AOP infarction was made.

## Discussion

In 1973, Gerard Percheron was the first person to describe the artery of Percheron (AOP). Between 4.7 % and 11.7 % of people have been said to have AOP (Kocaeli et al., 2013), but the actual rate of people who have it isn't clear because the current clinical routine method of intracranial vascular assessment can't make sure that it shows up. Previous studies showed that when the AOP was blocked, there was less acute ischemic cerebral infarction (Bordes et al., 2020).

## Anatomiacl base

The AOP is a single dominant artery of the thalamo-perforating artery, an uncommon cerebrovascular anatomical variant, which serves the contralateral paramedian areas of the thalamus and occasionally the rostral midbrain (Kuybu et al., 2021). Previous studies have found significant anatomical variations in the thalamoperforating artery in the healthy population. Lang and Brunner (Lang and Brunner, 1978) described four kinds of thalamus perforating artery patterns after examining 50 cadaver brains in 1978. Since then, a variety of classification schemes have been proposed based on their origin at the P1 segment, dividing them into 3–6 different types (Pedroza et al., 1986, Uz, 2007, Park et al., 2010). The classification scheme proposed by Uz in 2007 (Uz, 2007) is currently more widely accepted. According to the number of branches and the different sources of the unilateral or bilateral P1 segment, the thalamoperforating artery is divided into four types: type I (20 % of the subjects were bilaterally multiple): at least two or more branches of the thalamoperforating arteries were present on both sides; type II (unilateral multiple and unilateral single type accounted for 33 %): one side has multiple thalamoperforating arteries, the other side is not more than two thalamoperforating arteries; type III (bilateral unilateral for 40 %): only one thalamoperforating artery per side; and type IV (multiple on one side and no branch on the other side accounted for 7 %): no thalamoperforating artery on one side of the P1 segment, multiple thalamoperforating arteries on the other side, but this artery supplies bilateral paramedian thalamus area and rostral midbrain. The last mentioned artery is the Percheron artery. The proportion of each grade varies in different classifications. In an anatomical study of 34 formalin-fixed cadaveric brains, the thalamoperforating arteries were observed to have four types based on their origin at the P1 segment, including type I (55.8 %), type II (11.7 %), type III (20.5 %), and type IV (11.7 %) (Kocaeli et al., 2013). The observed variations included multiple arterial twigs forming the thalamoperforating artery, which arose from the superior or posterior surfaces of the P1 segment at an average distance of 1.87 mm from the basilar apex (Kocaeli et al., 2013). Similar to the aforementioned experiments, the last one also refers to AOP. Later, someone proposed that type II and type III be classified as type IIA and type IIb, and type IV as type III, with the result that AOP is also classified as type IIb.

## Clinical characteristics

The infarction resulting from the occlusion of the Percheron artery is called the AOP infarction, and the infarction sites are not always consistent. Lazzaro et al. (2010). looked at the imaging and clinical data of 37 patients with AOP infarction. They found four types of AOP infarction patterns: bilateral paramedian thalamic region infarction (43 %), rostral midbrain infarction (38 %), anterior thalamus and rostral midbrain infarction (14 %), and anterior thalamus without midbrain infarction (5 %). In this group of cases, patients in Cases 1 and 2 had midbrain involvement, while Case 3 only had bilateral paramedian thalamic involvement without midbrain or anterior thalamic involvement.

The three most common clinical symptoms in AOP infarction are disturbance of consciousness (94 %), gaze abnormalities (53 %), and memory impairment (24 %). In addition, extrapyramidal features (12 %) and headache (18 %) can also be present (Aaron et al., 2015). 94.2 % of patients with AOP infarction experience transient changes in consciousness, with about one-third experiencing disturbances of volatile consciousness. The duration of a sudden loss of consciousness is typically between 3 and 24 h (Aaron et al., 2015). In addition to ophthalmic symptoms, the typical symptoms of the regions involved in AOP obstruction are lowering of consciousness, memory impairment, vertical gaze paralysis, apathy, aphasia, cognitive dysfunction, dysarthria, and mental behavior abnormalities (Bordes et al., 2020). Other reported symptoms of AOP infarction include hemiplegia, cerebellar ataxia, motor disorders, seizures, tremors, fever, and headache (Aaron et al., 2015, Kheiralla, 2020, Musa et al., 2021). In the three cases presented in this study, all three patients experienced a decrease in their level of consciousness. In case 1, the female patient recovered consciousness within 2 h; in case 2, the male patient experienced mainly conscious fluctuation; and in case 3, the patient experienced sudden loss of consciousness. The duration of the loss of consciousness did not exceed 24 h. Approximately one-third of patients also show emotional indifference and inactive communication (Aaron et al., 2015). In this study, all three patients showed indifferent manifestations to varying degrees, with mostly simple answers when communicating with others.

53 % of people with AOP infarction have trouble moving their eyes up and down, 29 % have trouble moving their eyes up, and 12 % have trouble moving their eyes up and down (Aaron et al., 2015). In patients with paramedian thalamic infarction, about 84.8 % of neuro-ophthalmic dysfunction is oculomotor nerve dysfunction (Ranasinghe et al., 2020). In this study, case 1 had one eyelid that drooped and a smaller pupil, but the other two patients didn't show any obvious signs of neuro-ophthalmic dysfunction. 24 % of patients with AOP infarction had impaired memory, specifically anterograde amnesia. In this study, all three patients had varying degrees of memory loss during follow-up.

## Imaging assessment

During the hyperacute phase of a cerebral infarction, it is very important to find and treat a Percheron artery infarction as soon as possible. A clear diagnosis of Percheron artery infarction is usually made in the late stage, as imaging manifestations of the bilateral paramedian thalamus region and/or rostral midbrain ischemic infarction can only be fully visualized after the hyperacute phase. It can make it challenging to immediately identify the infarction position, leading to delays in diagnosis and management. However, there are no studies on the time from the onset of AOP infarction to the full display of infarction on DWI images. In this group of cases, the head MRI of Case 3 showed bilateral paramedian thalamus region lesions at 2 h after onset, while the MRI of Case 1 and CT of Case 2 did not fully display the infarction signals within 5 h after onset. Therefore, we recommend reviewing imaging if severe symptoms are highly suspected as AOP infarction despite the absence of acute infarction signs on a super-early head CT or MRI.

The brain MRI of typical AOP infarction patients showed symmetrical long T1 and long T2 signals, a Flair high signal in the bilateral paramedian thalamus, a high DWI signal, and a low ADC signal in the acute phase. As magnetic resonance imaging technology improves, apparent diffusion coefficient (ADC) images and diffusion weighted images have become the "gold standard" for diagnosing and treating AOP infarction, especially in the early stages. Lazzaro et al. (2010). found that FLAIR and DWI sequences in 67 % of patients with AOP infarction showed V-shaped high signal intensity on axial FLAIR and DWI images in the pial surface of the midbrain in the interpeduncular fossa. This showed that bilateral paramedian thalamus infarction was related to midbrain involvement. Aaron et al. (2015). said that 30 % of patients with AOP infarction who had FLAIR or T2-weighted images showed V signs of medial midbrain involvement. In this group of patients, within 2–5 h after onset, head CT failed to show a typical low-density signal in the paramedian thalamus. DWI showed restricted diffusion-limited signals in unilateral or asymmetric bilateral paramedian thalamus regions. The signals in bilateral paramedian thalamus regions were not fully displayed until 2–3 days after onset, possibly due to differential cellular tolerance to hypoxia and gradual involvement of blood supply areas through multiple small branches of AOP.

Conventional angiography, such as head MRA and CTA, may not be sufficient to determine whether AOP is the responsible vessel in the infarcted area due to the thinness of the vessel. Digital subtraction angiography may be a more effective technique for detecting the artery. In this group, Case 2 did not undergo cranial vascular imaging examination, while AOP was not detected in the MRA of Case 2 but was apparent in the CTA of Case 3. We recommend completing the DSA during follow-up to make up for this information gap.

It has also been reported that single photon emission computed tomography (SPECT) of cerebral perfusion can be utilized to assess the function of neuronal nuclei in the median parathalamic region (Wei et al., 2017).

## Risk factors

According to the Trial of Org 10172 in Acute Stroke Treatment (TOAST) classification system of cerebrovascular disease, the most common causes of AOP infarction are small-artery occlusion, lacunar bleeding, and cardioembolism (Hawkes et al., 2015). Some risk factors that affect AOP infarction include hypertension, diabetes, smoking, tumors, inflammation, coagulation disorders, hypotension, small vessel diseases, and cardiac embolism events (Kheiralla, 2020). Basilar tip aneurysms (BTA) and vertebral artery dissection (VAD) are also considered to be scarce risk factors for the AOP infraction (Kheiralla et al., 2021).

In our cases, Case 1 was considered to be cardioembolism (CE) brought on by atrial fibrillation emboli shedding without anticoagulation. The etiology of Case 2 was more complicated and classified as an acute stroke of other determined etiology (SOE), specifically considering that the patient had KD and also suffered from hypertension and diabetes. Therefore, it was considered that the etiology could not be excluded from large-artery atherosclerosis (LA) or small-artery occlusion lacunar (SAA). The cause of the Case 3 patient is ascribed to SAA, which is caused by impaired glucose tolerance.

## Differential diagnosis

Other diseases that can cause bilateral thalamic lesions besides AOP infarction are top of the basilar syndrome, deep venous thromboembolism, infections, demyelination, spongiform encephalopathy, thiamine deficiency, and hypoxic injury (Kheiralla, 2020). The most common diseases associated with bilateral thalamic lesions are: Top of the basilar syndrome: The affected areas are mostly located in the posterior circulation region, and multiple acute abnormal signals can be observed on brain imaging. These areas are distributed in the supply area of the posterior cerebral artery and superior cerebellar artery, such as the cerebellum, occipital lobe, brainstem, middle brain, unilateral thalamus, bilateral thalamus, medial temporal lobe, and occipital lobe (Kheiralla, 2020). Cerebral venous thrombosis: Bilateral thalamic involvement may occur, especially in cases involving Galen vein and straight sinus thrombosis. However, patients often exhibit clinical symptoms such as nipple edema, headache, epilepsy, and local nerve function defects (Kheiralla, 2020). Inflammatory diseases: Bilateral thalamic involvement may be observed in tuberculosis, malaria, meningitis, and viral infections, such as yellow fever virus and West Nile virus encephalitis. Clinically, these patients have specific exposure histories, fever histories, and special imaging features. Other diseases, such as infection, tumors, and rheumatism, can be excluded by examination (Lazzaro et al., 2010).

## Conclusion

Early imaging may have limitations in diagnosing the disease. Therefore, follow-up skull imaging is necessary to confirm the diagnosis if there are clinical symptoms. Patients with early consciousness decline and reduced communication should raise suspicion of AOP infarction.

## Ethics statement

The study was conducted in accordance with the principles of the Declaration of Helsinki, and the study protocol was approved by the ethics committee of Chinese PLA General Hospital. Because of the rtrospective nature of the study, patient consent for inclusion was waived.

## Funding

The author(s) received no financial support for the research, authorship, and/or publication of this article.

## CRediT authorship contribution statement

JL, JLG and GEY contributed the conception of the study and the clinical image analysis. JL and SHY contributed to MR data acquisition. JL contributed the clinical data acquisition. JL, JLG and GEY contributed to the interpretation and draft. JLG and GEY contributed to the revision for important intellectual content.

## Declaration of Competing Interest

The author(s) declared no potential conflicts of interest with respect to the research, authorship, and/or publication of this article.
